# Women’s self-care for Coronavirus prevention and the related factors in Iran: A cross-sectional study

**DOI:** 10.1371/journal.pone.0294983

**Published:** 2023-11-30

**Authors:** Soheila Nazarpour, Masoumeh Simbar, Farzaneh Rashidi Fakari, Mobina Khorrami, Khadijeh Dodel Andarvar, Zahra Jafari Torkamani, Sepideh Keyvanfar, Hamid Alavi Majd

**Affiliations:** 1 Department of Midwifery, Chalous Branch, Islamic Azad University, Chalous, Iran; 2 Midwifery and Reproductive Health Research Center, Shahid Beheshti University of Medical Sciences, Tehran, Iran; 3 Department of Midwifery and Reproductive Health, School of Nursing and Midwifery, Shahid Beheshti University of Medical Sciences, Tehran, Iran; 4 Department of Midwifery, School of Medicine, North Khorasan University of Medical Sciences, Bojnurd, Iran; 5 Department of Biostatistics, School of Paramedicine, Shahid Beheshti University of Medical Sciences, Tehran, Iran; The World Islamic Sciences and Education University, JORDAN

## Abstract

Promoting self-care can be an effective way to decrease the rate of transmission and expansion of the infection. However, there seem to be different related factors to self-care by gender. This study aims to assess women’s self-care for COVID-19 prevention and some related factors in Tehran-Iran in 2021. This was a descriptive cross-sectional study that was performed on 403 women living in Tehran in 2021. Subjects of the study were recruited using a multi-stage sampling method. Data was collected using a socio-demographic questionnaire as well as a valid and reliable questionnaire to assess women’s self-care for COVID-19 prevention (SCVP-38). The questionnaires were sent on the Google platform to the eligible participants. After completion of the forms, the created data in the Excel software in Google Drive were converted to SPSS 24 and then analyzed by using *t*-test, ANOVA, Pearson correlation, and multiple linear regression tests. The overall mean score of self-care was 71.84±17.81 percent. The highest and lowest scores were respectively related to using masks in public vehicles and not touching the face. Significant negative correlations were shown between the women’s self-care on Corona prevention with stress, anxiety, concerns, and fear (*P*<0.001). Linear multiple regression showed two factors including women’s age (p = 0.033) and their number of children (p = 0.042) predict the self-care, so that, for increasing each year to women’s age, SCVP increases by 0.463 units, and with increasing each child, SCVP decreases by 3.608 units. We concluded that in the COVID-19 pandemic, women in Tehran are performing more than 70% of measures of self-care. Also, education about self-care is recommended for younger women with more children. Moreover, promoting the self-care program can improve women’s mental health during the COVID-19 pandemic.

## Introduction

Coronavirus disease (COVID-19) is a global public health emergency and highly infectious new pneumonia caused by severe acute respiratory syndrome coronavirus 2 (SARS-CoV-2) [1]. It is an emerging infection that spread rapidly around the world [2] and it is now recognized as a health challenge worldwide [3]. The emergence of new types of coronavirus, which occurs after the high mutation of this virus, causes an increase in the ability to escape from the immune system and the high power of its contagion and spread [4]. Although many aspects of this disease are still unknown, its direct person-to-person transmission is confirmed [5] and asymptomatic individuals as a potential source of infection are identified [6]. COVID-19 still infects many people around the world and has spread widely in Iran. Symptoms of COVID-19 infection are mild and treatable in 80 percent of patients, but it may be severe and even fatal in others [7].

Promoting prevention measures is the most important strategy to control any epidemics of infectious and communicable diseases [8]. In this accordance, community education about self-care and taking responsibility about own and others’ health are the prerequisites for the successful implementation of a program [9]. By the way, using disease prevention and management techniques, improving lifestyle, teaching health tips, and providing self-care guidelines can help prevent future outbreaks of diseases [10]. A key strategy to combat COVID-19 transmission is self-care promotion [11] which can decrease the risk of its transmission [7, 10] and individuals should pursue self-care strategies as long as there is the risk of transmission [10].

Self-care as a theory focuses on the practice of activities that individuals initiate and perform on their behalf to maintain life, health, and well-being. Dorothea Orem (self-care requisites and self-care behaviors) and Jean Watson (self-compassion within the human caring theory) were among the pioneers of self-care theory [12, 13]. World Health Organization (WHO) defines self-care as “the ability of individuals, families, and communities to promote their health, prevent disease, maintain health, and to cope with illness and disability with or without the support of a health worker" [14]. Self-care is a practice in which everyone uses their knowledge, skills, and abilities as a resource to improve their health status independently. The key concepts of self-care are maintenance, self-care monitoring, and self-care management. In addition, self-care guidelines can be used as practical ways to slow the progression of diseases and improve quality of life [15]. Self-care is considered a significant and valuable strategy as it emphasizes the active role of individuals in maintaining their health and well-being. Today, self-care is considered a key component of health promotion to improve the health, and well-being of individuals and as a strategy to reduce the high cost of medical services [16, 17].

Self-care existed before formal health systems and plays an important role in health outcomes. For many people in some cultural and social backgrounds, self-care is an established behavior. Self-care is an important component of an individual’s health even if there is access to a health care system. In addition, interventions that were previously only available through healthcare providers, are now being accessed in the self-care setting [18]. Individuals can solve their health problems through self-care and by raising awareness and modifying their lifestyle [19] Self-care is important in all aspects of health and it is particularly essential for populations who are negatively affected by sex, political, cultural, and power dynamics such as women [18]. Self-care assessment is a prerequisite for self-care promotion interventions [20].

In the recent coronavirus epidemic crisis, promoting self-care is a key strategy to prevent COVID-19 prevention and also to enhance physical and mental health [18], and it is now widely recommended to practice self-care to reduce stress and the risk of COVID-19 infection and the complications [21, 22]. Given the importance of self-care for COVID-19 prevention for the control and management of the COVID-19 epidemic crisis [10], it is important to evaluate the self-care behavior of individuals. For instance, self-care during the COVID-19 pandemic was assessed in Mixico-City and showed 66 to 80% complied with self-care recommendations [11], Many studies in Iran and other countries are concentrated on reproductive health self-care [23–27]. However, to our knowledge there were no study assessed women’s self-care for Coronavirus (COVID-19) prevention and its related factors.

Gender differences in health behavior during the epidemic have not yet been fully elucidated. [28]. However, there seem to be different responses to health and self-care measures by country, age, and gender [29]. The results of studies show that women in these conditions are more at risk of mental consequences such as stress, anxiety, and depression [29, 30]. Besides, women are generally more involved in self-care activities and adopt healthier daily routines than men [9, 29].

Especially married women are usually the center of the family and take responsibility for maintaining the family members’ health comfort, and tranquility. This can be attributed to the women’s biological characteristics and the social expectations about women’s gender roles as wives or mothers. The consequence of such an experience is that they use their maximum capacity to care for and protect the family during the COVID-19 epidemic despite the stressful effects of the crisis [9]. Women are more likely than men to be cautious about the risks of the coronavirus pandemic and to provide more protection, including hand washing, using face masks, and sanitizing surfaces [31]. Therefore, considering gender differences in self-care studies can lead to more accurate results. Therefore, the present study aimed to assess women’s self-care for Coronavirus (COVID-19) prevention and the related factors in Tehran.

## Methods

### Study design

This was a descriptive cross-sectional study.

### The particiants

The study was performed on women aged more than 18 years old, and residents in Tehran with no known medical conditions or psychological disorders. In this study, the psychological status of participants was considered in the last six months. They were contacted by the health providers of the selected health centers and following the explanation about the aims and procedure of the study, if they accepted to participate informed online consent was obtained. It should be emphasized the participants’ online consent was required for the completion of the form and they could not access the questionnaire if they did not give the consent.

### Sampling method

The women were recruited using a multi-stage cluster sampling method. First, health centers affiliated with Shahid Beheshti University of Medical Sciences were listed from 4 geographical areas of Tehran, and then 5 centers in each area were randomly selected using a quota sampling method and based on the client population of each center the sample size was calculated and then the eligible women were selected. After contacting them, the Google form link was sent to them to complete. The link to the online questionnaire was sent to the participants by electronic message or WhatsApp, following a cellphone call to them and explaining the goals and process of the study as well as obtaining verbal informed consent. Then electronic written informed consent was also obtained from all participants and completing the forms was only possible after giving the informed consent of the participant. For women who were not familiar with the electronic Google forms, the questionnaire was filled out by the researcher through a telephone interview.

### Sample size

The minimum sample size was calculated using the formula of descriptive studies and considering the 50% probability of women’s self-care the Type I error of 0.05 and the absolute error of 0.5, the minimum sample size of 385 samples was calculated.


$$n\geq \frac{z_{1-\alpha /2}^{2}(1-P)}{\varepsilon^{2}P}$$

### Tools for data collection

Two questionnaires were used for data collection including (1) a personal and socio-demographic characteristics questionnaire (2) a questionnaire to assess self-care for Coronavirus (COVID-19) prevention (SCVP-38) and (3) a self-assessment scale to measure women’s mental status.

#### (1) Personal and socio-demographic characteristics questionnaires

This questionnaire contained 17 questions about the personal, social, economic, and anthropometric characteristics of participants including age, weight, height, education, and occupation of women, marital status, employment and education of the spouse if married, adequacy of income, housing status, number of children, medical history and condition, history of Corona infection, vaccination against coronavirus (including the vaccine type and complications) and the impact extent of Coronavirus (COVID-19) pandemic crisis on women’s mental status, including 5 items to assess, stress, anxiety, depression, fears, and concerns, scoring from 1 to five for never to always scale. The validity of the questionnaire was assessed by 10 faculty members of Shahid Beheshti University of Medical Sciences.

#### (2) Self-care for Coronavirus (COVID-19) prevention (SCVP-38) questionnaire

This questionnaire consisted of 38 questions with 1 to 5 scores for never, rarely, sometimes, often, and always responses, respectively. The scores range between 38 to 190. Higher scores from the questionnaire showed healthier self-care. To standardize, the total scores were calculated from 0 to 100 based on the following formula.

(X-Min Score / Max-Min Score) × 100

This questionnaire was developed using a deductive approach and based on a review of the guidelines of the World Health Organization [32], CDC [33], Iranian Ministry of Health [34], and Iranian Association of Health Education and Health Promotion [35]. The content validity of the questionnaire was assessed by 12 experts in Public Health, Reproductive Health, and nurses.

The face validity of the questionnaire was assessed by impact score calculation. All items of the questionnaire had a score of more than 1.5 and were considered important by the participants. The results showed minimum to maximum impact scores of 3.25 to 4.85. The content Validity of the questionnaire was assessed by calculating the Content Validity Ratio (CVR) and Content Validity Index (CVI). CVR ranged from 0.83 to 1. The modified content validity index of I-CVI for all items ranged from 0.91 to 1, and the S-CVI / Average score was 0.99.

The reliability of the questionnaire was measured by calculating Cronbach’s alpha coefficient for internal consistency assessment and also calculating the Pearson coefficient to measure the stability of the questionnaire by the test-retest method. The results showed the reliability of the questionnaire by α = 0.98 and Pearson r = 0.98.

#### (3) Self-assessment scale to measure women’s mental status

This scale was designed to assess the impact extent of the Coronavirus (COVID-19) pandemic crisis on women’s mental status. It contained 5 items to assess, stress, anxiety, depression, fears, and concerns, scoring from 1 to five for never to always scale. The validity of the questionnaire was assessed by 10 faculty members of Shahid Beheshti University of Medical Sciences. Test-retest on 15 women was used to assess the reliability of the scale and a Pearson correlation of 0.98 showed the reliability.

### Statistical analysis

After filling out the Google forms by the participants in the Google platform, the data were generated in the Excel program in Google Drive. Then the data in the Excel file was converted to SPSS. Thereafter the data were analyzed using SPSS-24 and by t-test, ANOVA, Pearson correlation coefficient tests, and linear multiple regression analysis. T-test was used to compare two groups of quantitative variables with normal distribution, and the Kolmogorov-Smirnov test was used to check normality. P values less than 0.05 were considered statistically significant.

### Ethics approval and consent to participate

The study was approved by the ethics committee of Shahid Beheshti University of Medical Sciences, with the code “IR.SBMU.RETECH.REC.1399.584”. All methods were performed according to the relevant guidelines and regulations as approved by the deputy of research and the ethical committee of Shahid Beheshti University of Medical Sciences. Online informed consent was obtained from all participants after explaining the objectives and procedure of the study.

## Results

Four hundred three women aged 31.58 ± 9.41 (Mean ± SD) years, with the range 18 to 80 years participated in the study. The majority of the participants (97.3%) were urban residents and the rest were rural residents. The demographic characteristics of the participants are shown in Table 1.

**Table 1:** Socio-demographic characteristics of the women (n = 403).

**Variables**		**Mean ± SD**
Age (years)		31.58±9.41
Weight (kg)		67.38±12.29
Height (cm)		164.31±6.35
BMI (kg/m^2^)		25.00±4.60
Number of children		1.17±1.24
		**N (%)**
Residency	City	392 (97.3)
	Village	11 (2.7)
Marital Status	Single	97 (24.1)
	Married	286 (71.0)
	Widow/ Divorced	20 (5.0)
Education	Under diploma	32 (7.9)
	Diploma	72 (17.9)
	Associate degree/ Bachelor	209 (51.9)
	Masters/ Doctorate	90 (22.3)
Education of husband	Under diploma	23 (5.7)
	Diploma	70 (24.2)
	Associate degree/ Bachelor	129 (32.0)
	Masters/ Doctorate	67 (16.6)
Occupation	Employed	109 (27.0)
	Housewife	208 (51.6)
	Student	86 (21.3)
Husband Occupation	Employee	112 (38.4)
	Manual worker	18 (6.2)
	Freelance	151 (51.7)
	Retired/ Unemployed	11 (3.8)
Housing situation	The owner	199 (49.4)
	Rent or mortgage	167 (41.4)
	Relatives’ home	37 (9.2)
Family income	Inadequate	201 (49.9)
	Adequate	130 (32.3)
	Adequate and savings	72 (17.9)

**Fig 1:**
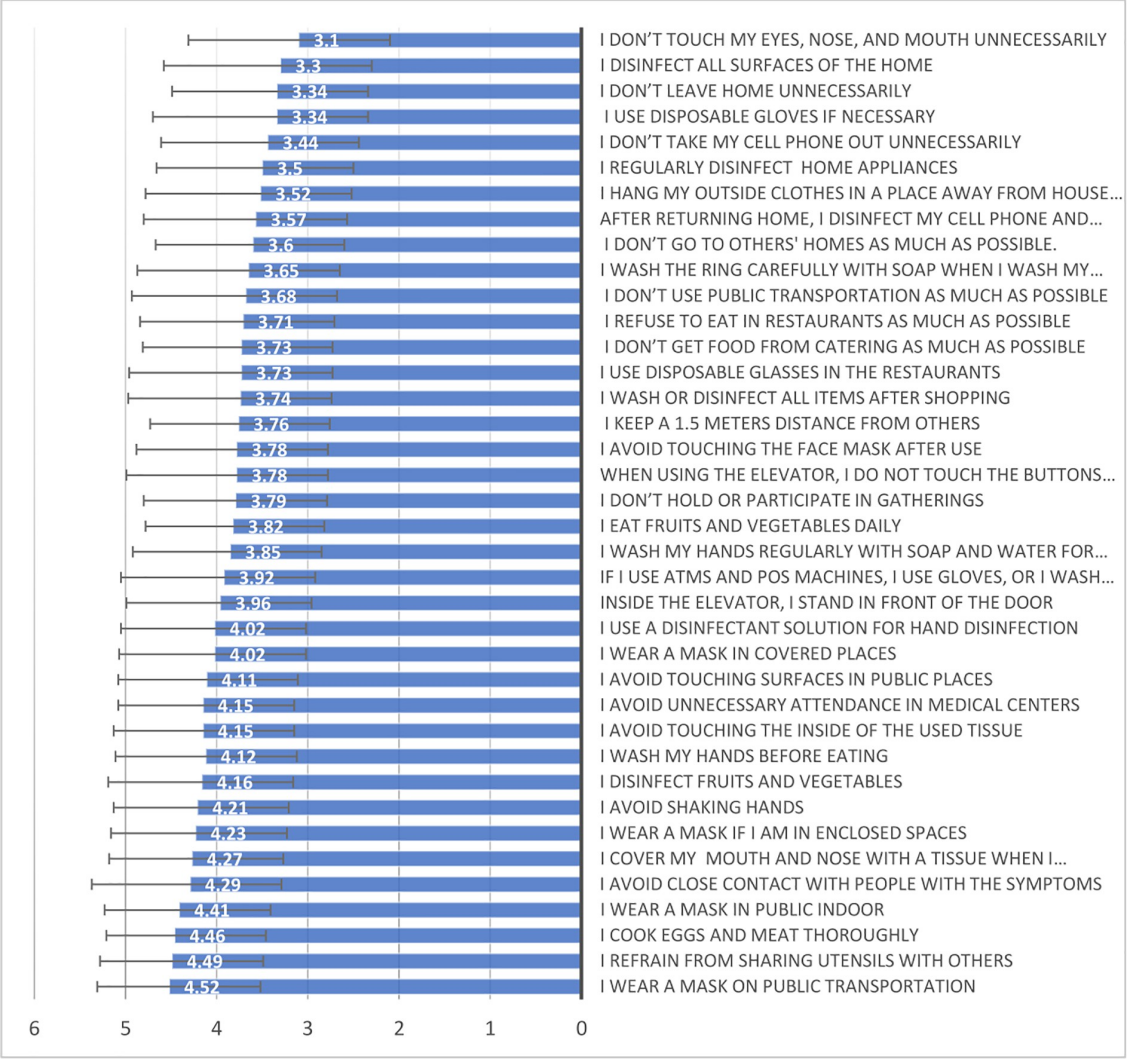
Mean (±SD) of women’s self-care scores for Coronaviruses prevention behaviors (WSCPB).

Results showed a self-care score of 71.84±17.81 Percent. The items with the highest and lowest scores were “the use of masks in public vehicles” and “not touching the face (eyes, nose, and mouth) under any circumstances”, respectively (Fig 1).

Table 2 shows the relationship between women’s self-care in Coronavirus prevention with the participants’ socio-demographic characteristics. The results demonstrated no relationship between self-care and the socio-demographic characteristics of women.

**Table 2:** The relationship between women’s self-care for Coronavirus prevention with socio-demographic characteristics of women.

Demographic factors	WSCPB Score	
	*r/t/F* ^a^	*P* value
**Age**	*r* = 0.088	0.078
**BMI**	*r* = -0.021	0.677
**Number of children**	*r* = -0.004	0.937
**Residency**	*t =* 1.632	0.103
**Marital Status**	*F* = 2.136	0.119
**Education**	*F* = 1.579	0.207
**Education of husband**	*F* = 2.023	0.134
**Occupation**	*F* = 2.284	0.059
**Job of Husband**	*F* = 0.947	0.437
**Housing situation**	*F =* 0.376	0.687
**Family income**	*F* = 0.918	0.400

^a^Test: *r*: Spearman’s correlation; *t*: Independent sample *t*-test, *F*: ANOVA

Table 3 shows the frequencies and mean scores of women’s self-care for Coronavirus prevention of women with or without a medical condition. The results also demonstrated no significant difference between women’s self-care on Corona prevention with (72.74 ±15.86) or without medical conditions (71.35±18.81) (*t-*test; p = 0.454).

**Table 3:** The frequencies and mean scores of women’s self-care on Coronavirus prevention of women with or without a medical condition.

Medical Condition	N (%)		Mean (SD)	
	Without medical condition	With medical condition	Without medical condition	With medical condition
Medical problems	260 (64.5)	143 (35.5)	71.35 (18.81)	**72.74 (15.86)**
Cardiovascular diseases	377 (93.5)	26 (6.5)	71.52 (17.89)	**76.59 (16.07)**
Pulmonary diseases	399 (99.0)	4 (1.0)	71.71 (17.83)	**85.20 (7.68)**
Urinary Tract diseases	394 (97.8)	9 (2.2)	71.89 (17.81)	**69.95 (18.54)**
Gastrointestinal diseases	383 (95.0)	20 (5.0)	71.80 (18.3)	**72.63 (13.4)**
Diseases of the liver, gallbladder, and pancreas	396 (98.3)	7 (1.7)	71.73 (17.84)	**78.38 (15.46)**
Diabetes	382 (94.8)	21 (5.2)	72.23 (17.48)	**64.72 (22.35)**
Thyroid disease	348 (86.4)	55 (13.6)	71.65 (18.24)	**73.10 (14.87)**
Musculoskeletal diseases	397 (98.5)	6 (1.5)	71.87 (17.91)	**69.96 (9.20)**
Psychological diseases	384 (95.3)	19 (4.7)	71.78 (17.77)	**73.03 (18.94)**
Cancer	399 (99.0)	4 (1.0)	71.70 (17.81)	**85.85 (12.51)**
Dermatological diseases	490 (96.8)	13 (3.2)	71.82 (18.00)	**72.47 (10.69)**
Other	379 (94.0)	24 (6.0)	71.76 (17.89)	**73.14 (16.74)**

However, the results revealed a significant negative correlation between the women’s self-care on Corona prevention with their mental status including the level of stress, anxiety, concerns, and fear, respectively. Analysis of variance also confirmed the results (Table 4).

**Table 4:** The correlations between women’s self-care Coronavirus prevention and their mental status.

Mental status		Mean±SD	ANOVA		Spearman Correlation	
			*F*	*P*	*r*	*P*
**Depression**	Never	74.49±14.63	1.506	0.200	-0.071	0.154
	Low	74.23±14.76				
	Medium	71.95±17.13				
	Much	70.03±18.20				
	Very much	68.62±23.17				
**Anxiety**	Never	82.76±10.66	11.139	<0.001	-0.290	<0.001
	Low	75.92±14.64				
	Medium	70.84±16.57				
	Much	66.01±17.44				
	Very much	65.27±24.42				
**Fear**	Never	79.80±13.42	8.759	<0.001	-0.257	<0.001
	Low	76.45±14.63				
	Medium	71.72±16.11				
	Much	65.25±18.70				
	Very much	65.76±24.90				
**Stress**	Never	82.88±10.70	12.915	<0.001	-0.320	<0.001
	Low	75.81±15.29				
	Medium	70.56±16.29				
	Much	64.52±18.34				
	Very much	64.78±24.67				
**Concern**	Never	80.42±14.37	9.574	<0.001	-.0.263	<0.001
	Low	73.84±14.18				
	Medium	70.72±16.24				
	Much	66.62±19.10				
	Very much	60.89±28.42				

The results also demonstrated no significant difference between SCVP of women who were affected with COVID-19 or not affected, and performed or not performed vaccination (Table 5).

**Table 5:** The comparison of self-care for Coronavirus prevention of women who were affected by COVID-19 or were not affected and performed or not performed vaccination.

**Variables**		**Self-Care of Covid-19**		
		**N (%)**	**Mean±SD**	**t-test (P)**
**History of Corona**	Yes	196 (48.6)	147.05±25.39	0.913
	No	207 (51.4)	147.35±28.63	
**COVID19 vaccine injection**	Yes	247 (61.3)	147.87±27.22	0.537
	No	156 (38.7)	146.15±26.89	
		**N (%)**	**Mean±SD**	**ANOVA (P)**
**Type of vaccine**	Covaxin (Indian)	12 (4.9)	132.67±27.17	0.076
	AstraZeneca (Korean)	50 (20.2)	147.80±24.13	
	Sinopharm (Chinese)	149 (60.3)	150.21±27.35	
	Sputnik V (Russian)	32 (13.0)	145.88±27.05	
	Other	4 (1.6)	122.75±44.54	

Linear multiple regression test showed that the model is predicting 4.1% of the SCVP Besides, two factors including women’s age (p = 0.033) and their number of children (p = 0.042) predict SCVP (Table 6). So, for increasing each year to women’s age, SCVP increases by 0.463 units, and with increasing each child, SCVP decreases by 3.608 units.

**Table 6:** Linear regression of possible predictive factors related to self-care for coronavirus prevention in women participating in the study.

Variables	B	Std. Error	Beta	t	*P*
**Age**	0.463	0.216	0.161	2.143	0.033
**BMI**	-0.566	0.341	-0.096	-1.660	0.098
**Marital Status**	4.832	3.808	0.090	1.269	0.205
**Number of Children**	-3.608	1.769	-0.165	-2.039	0.042
**Family income**	0.701	1.786	0.020	0.392	0.695
**Housing situation** ^**b**^	-1.619	2.124	-0.039	-0.763	0.446
**Medical problems** ^**c**^	-2.305	2.944	-0.041	-0.783	0.434
**Education** ^**d**^	0.038	1.194	0.002	0.032	0.975
**Occupation** ^**e**^	3.906	3.050	0.072	1.281	0.201
**R Square = 0.041 Adjusted R Square = 0.019** ^**f**^					

^a^Classification of family income: 1. Inadequate, 2. Adequate, 3. Adequate and savings

^b^Classification of housing situation: 1. Owner, 2. Rent or mortgage, 3. Relatives’ home

^c^Classification of medical problem: 0 (zero). None, 1.Yes

^d^Classification of education:1. Illiterate, 2. Under diploma, 3. Diploma, 4. Associate degree, 5. Bachelor, 6. Masters, 7. Doctorate

^e^Classification of occupation: 1.Employed, 2. Housewife or retired.

^f^ R-squared (R2) represents the proportion of the variance in the dependent variable explained by the independent variables. Adjusted R-squared considers the number of predictors in the model and penalizes excessive variables, providing a more accurate measure of the model’s goodness of fit, especially with multiple predictors. The closer these values are to 1, the more the model expresses the relationship between the dependent and independent variables.

## Discussion

This study assessed the related factors with women’s self-care on Coronavirus prevention in Tehran and found that women had the highest score for the behavior of "using a mask in public transportation" and the lowest self-care score for the item of “not touching the eyes, nose and mouth”. There were also significant negative correlations between women’s self-care on Corona prevention and their mental status including the levels of anxiety, fear, stress, and anxiety. Also, women’s age and number of children predict the level of their self-care in Covid-19 prevention.

The results showed that women in Tehran meet most of the criteria recommended by the valid sources for self-care on COVID-19 prevention. It seems community education about self-care on COVID-19 prevention through digital resources and social media plays a significant role in promoting self-care behaviors. Besides, sharing health information from reliable scientific sources is one of the most effective strategies for promoting self-care in COVID-19 prevention and mental health enhancement of people during the COVID-19 pandemic crisis [36].

The results indicated the highest score for the behavior of “using a mask in public vehicles" and then "refusing to use shared utensils with sick people." Given that these are the basic principles for preventing respiratory infectious diseases, it seems that community education about Coronavirus prevention and self-care led to raising awareness [37] among women.

The lowest scores were related to the participants’ behaviors about "not touching their face including their eyes, nose and mouth” and then “disinfection of all surfaces of the home with alcohol spray". It seems self-care education is necessary for all items; however, more emphasis is essential on community health education through the media about “not touching eyes, nose and mouth”, “disinfecting high-touching surfaces and tools”, and “not leaving home for unnecessary reasons during COVID-19 epidemic”.

The present study showed that age can be a predictor of women’s self-care in COVID-19 prevention. This result is consistent with the results of similar studies that showed middle-aged women pay more attention to self-care during the COVID-19 epidemic and adopt healthier daily routines [9, 29]. By passing through life, people’s experiences of adaptation and resilience in different situations develop, which may explain healthier and more protective behaviors of middle-aged women compared to younger women [9]. A study found that people who perceived greater risks were more likely to engage in protective behaviors [38]. It seems perceived the risk improves with aging, which causes taking more responsibility for protecting and caring about themselves and their family members.

The findings showed that self-care for the prevention of the coronavirus among women with or without a medical condition is not significantly different. Although some studies found that people with medical conditions such as cancer, cardiovascular or pulmonary diseases have more self-care and seek more prevention methods [39–41] the measures as well as the tools for measurement of self-care for the prevention of the coronavirus are different from the prevention of other medical conditions [23, 42, 43].

The regression analysis in the present study showed the number of children could also be a potential predictor of women’s self-care for Coronavirus prevention. Bahrami and colleagues found a correlation between knowledge about self-care and the number of children [44]. It seems that women with more children find less time and opportunities for self-care measures. Besides, more children may increase parental stress and responsibility as well as financial problems [45]. A study showed more children are associated with lower quality of life, which was attributed to their greater responsibilities in large families. The numerous responsibilities of women in large families can leave them with less time and opportunity to perform self-care measures [46].

The findings of the study showed that women evaluated the effect of the coronavirus pandemic on their mental status on increasing depression, anxiety, fear, stress, and anxiety, respectively. The outbreak of COVID-19 caused many countries to make mobility regulations for citizens to reduce the potential risk of infection. Quarantine and the restrictions have affected people’s lifestyles and caused mental health consequences during and after the COVID-19 outbreaks [47, 48]. According to these studies, the negative impact of the COVID-19 crisis and its mental consequences is very wide. A study conducted by an online survey during the COVID-19 epidemic found symptoms of severe anxiety and severe depression in the general population among 20.8% and 27.5% of the general population, respectively [11]. According to some studies, the most important negative mental consequences of quarantine in the COVID-19 crisis are anxiety and distress [49, 50]. Similarly, other mental problems such as increased feelings of loneliness, stress disorders and post-traumatic stress symptoms, anger, fear, and sleep disorders are reported in the literature [11, 47, 51, 52]. Therefore, promoting self-care measures is an important factor in preventing immediate and late consequences of the COVID-19 pandemic [53].

The results indicated the significant negative correlations between self-care for Coronavirus prevention with women’s evaluation of the impact of COVID-19 on their mental status including depression, anxiety, fear, stress, and worries. Recent evidence demonstrated women are reporting higher scores on perceived stress than men during lockdown which is attributed to women’s roles, mainly the burden of both work and caring roles. An overload of women’s caring roles and an experience of greater stress levels and mental health problems contribute to a greater vulnerability for women and therefore, they are more likely than men to avoid healthy behaviors and self-care [29, 54]. Self-care education is one of the dimensions of psychological empowerment to prevent infectious diseases such as coronavirus prevention [36]. Self-care is considered a significant strategy for keeping an individual’s psychological well-being because it significantly operates as a mediating mechanism in the association between perceived stress and mental health in a quarantine situation [47].

Consistent with our results another study found that self-care decreased with increasing fear of COVID-19 [55]. Mental problems such as anxiety, fear, stress, and worries seem to cause stress that can be an obstacle to self-care and protection.

Finally, the tool, method, and findings of the present study are recommended to be used by the researchers and policymakers to show the need for interventional programs and planning an evidenced-based coronavirus prevention promotion program.

### Strengths and weaknesses

This study has several salient features that make it stand out from other studies. The study has focused on three main and significant recent subjects including (1) self-care which has been an important focus of the World Health Organization in recent years [14], (2) Coronavirus (COVID-19) which is the topic of the day, and (3) women’s health, which is a requirement of the development program [56]. The other strength of the study is the use of valid and reliable questionnaires.

One limitation of the study was the use of an electronic self-report questionnaire that may be less accurate than written questionnaires. An attempt was made to increase the accuracy of completing the questionnaires by training and sending instructions to colleagues and participating women.

Another limitation of this study is that, in addition to the history of corona, hospital admission was an important issue to be evaluated. However, it is not considered in the present study.

## Conclusion

During the coronavirus pandemic, women are practicing more than seventy percent of the self-care measures for coronavirus (COVID-19) prevention in Tehran. Considering that age can be a potential predictor of self-care, it seems appropriate self-care educational interventions for young women are necessary. Also, regarding the results that increasing the number of children can be a potential negative predictor for women’s self-care for COVID-19 prevention, it seems mothers who are busy with many children need more easily to access health facilities, education, and reminders to improve their self-care and family care.

Regarding the negative relationship of self-care with mental status such as increased depression, anxiety, fear, stress and worry during the coronavirus pandemic, education and promotion of self-care measures are suggested. Besides, community education, especially women’s education about copying strategies during the pandemic crisis is emphasized. Self-care promotion can help to improve people’s especially women’s mental health during the COVID-19 pandemic crisis.

Promoting self-care is strongly recommended in public health, not only in the prevention of COVID-19 but also in the prevention of all epidemic respiratory viral infections. Besides self-care promotion is particularly necessary for women who take the main role of family care. The effectiveness of the promotion programs is also essential using a valid tool like the one introduced in this study for improving future programs.

## Supporting information

S1 Checklist STROBE statement—checklist of items that should be included in reports of observational studies. (DOC)

S1 File (SAV)
